# Identifying critical edges in complex networks

**DOI:** 10.1038/s41598-018-32631-8

**Published:** 2018-09-27

**Authors:** En-Yu Yu, Duan-Bing Chen, Jun-Yan Zhao

**Affiliations:** 1 Big Data Research Center, University of Electronic Science and Technology of China, Chengdu, 611731 P. R. China; 2 The Center for Digitized Culture and Media, University of Electronic Science and Technology of China, Chengdu, 611731 P. R. China; 3 Beijing Special Vehicle Institute, Beijing, 100072 P. R. China

## Abstract

The critical edges in complex networks are extraordinary edges which play more significant role than other edges on the structure and function of networks. The research on identifying critical edges in complex networks has attracted much attention because of its theoretical significance as well as wide range of applications. Considering the topological structure of networks and the ability to disseminate information, an edge ranking algorithm *BCC*_*MOD*_ based on cliques and paths in networks is proposed in this report. The effectiveness of the proposed method is evaluated by SIR model, susceptibility index S and the size of giant component *σ* and compared with well-known existing metrics such as Jaccard coefficient, Bridgeness index, Betweenness centrality and Reachability index in nine real networks. Experimental results show that the proposed method outperforms these well-known methods in identifying critical edges both in network connectivity and spreading dynamic.

## Introduction

The structure and function of complex networks attracted a great deal of attention in many branches of science^1^. Networks mediate the spread of information, sometimes, a few initial seeds can affect large portions of networks. Such information cascade phenomena are observed in many situations, for example, cascading failures in power grids, diseases contagion between individuals, innovations and rumors propagating through social networks, and large grass-roots social movements in the absence of centralized control. How to find critical nodes and edges is an important and interesting issue. With the rapid development of internet media, the information interaction between individuals is becoming more and more frequent and the mechanism of information diffusion has become more and more complex. Many methods are used to measure the importance of nodes in networks. Degree centrality^2^, semi-local centrality^3^, *k*-shell^4^ and H-index^5,6^ are based on nodes’ degrees. Closeness centrality^7^, betweenness centrality^8^ and eccentricity centrality^9^ are based on paths in networks. PageRank^10^, LeaderRank^11^ and HITs^12^ are based on eigenvector. Sleep scheduling^13^ is one of the approaches to save residual energy of wireless nodes in energy-constraint large-scale industrial wireless sensor networks while satisfying network connectivity and reliability. In comparison, critical edges also play a significant role in the process of information diffusion. In complex networks, sometimes it is impractical to forbid all communications of a node, so it is necessary to truncate some important communication links. Critical edges analysis will be beneficial to guide or control the information dissemination from a global perspective.

In order to explore the transmission of information, many researches have focused on the network topology to find the critical edges. Degree product^14^ supposes that edges connecting two nodes with high degrees are critical. Betweenness centrality of edges^15,16^ and betweenness centrality of a group of edges^17^ suppose that edges linking two connected components are important. Average node reachability and the maximum flow of a network can characterize the ability of information transmission in networks and critical edges have serious influence on average node reachability and maximum flow^18,19^. In Jaccard coefficient^20^, if node *i* and node *j* have a lot of common neighbors, even if they have no direct connection, information also can spread from node *i* to node *j* easily, so edges are more important if there are less common neighbors. Complex networks may have many cliques. In Bridgeness^21^, if an edge is removed, information can spread through other edges in the clique which contains the removed edge, so, intuitively, edges in smaller cliques are more important.

What’s more, The ability to disseminate information is also an evaluation index to measure the importance of edges. In online social networks, the study finds three different spreading mechanisms: social spreading, self-promotion and broadcast^22^. An edge is important if most of the information is spreading through this edge^23^.

In this report, we only use the topology of networks to rank the importance of edges, considering not only the local characteristics (degrees of nodes, cliques) but also the global characteristics (betweenness centrality). The proposed method is compared with Jaccard coefficient, Bridgeness, Betweenness centrality and Reachability index in three evaluation metrics, SIR model^24,25^, susceptibility index *S*^26^ and the size of giant component *σ*^27^ in nine real networks which have large differences in basic topological features and the results show that the proposed method in this report can quickly decompose networks and has a greater impact on information spreading.

## Results

If there are many different cliques containing two related nodes of an edge, the edge is not so important for the perspective of spreading. Based on above point and betweenness centrality of edges, a new index *BCC*_*MOD*_ (Betweenness Centrality and Clique Model) is proposed to measure the importance of an edge *e*(*u*, *v*). *BCC*_*MOD*_ is an index which combines the local and global characteristics. In *BCC*_*MOD*_, if we remove edges with high score, the effect of spreading is large. The performance of *BCC*_*MOD*_ is compared with that of Jaccard coefficient, Bridgeness, Reachability and Betweenness. The results show that *BCC*_*MOD*_ can quickly decompose networks and has a greater impact on information spreading in most cases comparing with other methods. The detailed definitions of indices are given in the Method section.

### Data Description

Nine undirected and unweighted networks are used to evaluate the performance of the edge ranking method. (1) Jazz, a collaboration network between Jazz musicians. (2) Oz, a network contains friendship ratings between 217 residents living at a residence hall located on the Australian National University campus. (3) Highschool, a network contains friendships between boys in a small high school in Illinois. (4) Innovation, a network spread among 246 physicians in five towns, i.e., Illinois, Peoria, Bloomington, Quincy and Galesburg. (5) Lesmis, a network contains co-occurances of characters in Victor Hugo’s novel Les Miserables. (6) Train, a network contains contacts between suspected terrorists involved in the train bombing of Madrid on March 11, 2004 as reconstructed from newspapers. (7) PowerGrid, a network contains information about the power grid of the Western States of the United States of America. (8) Email, a network contains the email communication at the University Rovira i Virgili in Tarragona in the south of Catalonia in Spain. (9) Router, a network contains autonomous systems of the Internet connected with each other. All data can be downloaded from Chicago network dataset^28^ and the basic topological properties of these nine networks are shown in Table 1. In order to guarantee the diversity of networks, these nine networks have large differences in total number of nodes and edges, average degree, maximum degree, average clustering coefficient and degree heterogeneity.

**Table 1:** The basic topological features of nine real networks.

Networks	*n*	*m*	〈*k*〉	*k*_*max*_	*c*	*H*
PowerGrid	4944	6596	2.6682	19	0.0800	1.4505
Lesmis	77	254	6.5974	36	0.5731	1.8272
Router	5022	6258	2.4922	106	0.0116	5.5031
Jazz	198	2742	27.6970	100	0.6175	1.3951
Email	1133	5451	9.6222	71	0.2202	1.9421
Innovation	244	925	7.5819	28	0.3077	1.2764
Train	67	245	7.3134	29	0.5944	1.7100
Highschool	73	276	7.5616	19	0.4458	1.2242
Oz	217	1839	16.9493	56	0.3627	1.2094

*n* and *m* are the total number of nodes and edges, respectively. 〈*k*〉 is the average degree for networks. *k*_*max*_ is the maximum degree for networks. 〈*c*〉 is the average clustering coefficient and *H* is the degree heterogeneity, defined as $H=\frac{\langle {k}^{2}\rangle }{{\langle k\rangle }^{2}}$.

### Evaluation metrics

Susceptibility index *S*, the size of giant component *σ* and SIR spreading model are used to evaluate the performance of ranking methods.

#### Susceptibility index S

In network connectivity metric, susceptibility index *S* is used to evaluate the performance of methods. Susceptibility index *S* is defined as:

1 $$S=\sum _{s < {s}_{{\rm{\max }}}}\frac{{n}_{s}{s}^{2}}{n},$$

where *n*_*s*_ is the number of components whose size equals *s*, *s*_max_ is the size of giant component, and *n* is the size of whole network. For details, sort edges in descending order according to their ranking score firstly, and then calculate the Susceptibility index *S* after removing the edges from network one by one from high to low ranking scores. In this report, parameter *p* is defined as:

2 $$p=\frac{{m}_{r}}{m},$$

where *m* is the number of all edges and *m*_*r*_ is the number of removing edges.

**Figure 1:**
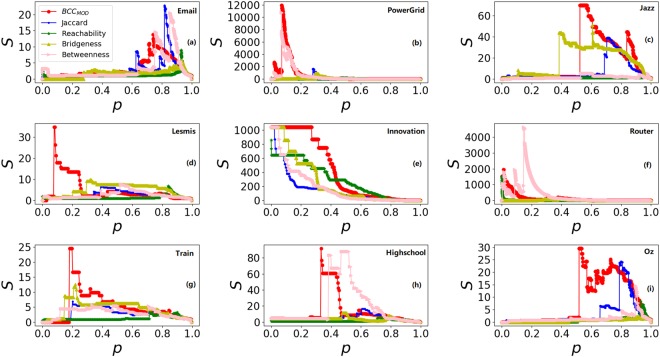
The susceptibility index *S* over different value of *p*.

The results are shown in Table 2 and Fig. 1. From Table 2 and Fig. 1, it can be seen that *BCC*_*MOD*_ has the minimum *p* when the largest *S* achieves in Lesmis, Highschool, Jazz, Train, Email and Oz. In Innovation, all methods have the same effect. In PowerGrid and Router, the largest *S* of *BCC*_*MOD*_ is appeared the second earliest. So, the largest S of *BCC*_*MOD*_ appeared the earliest in most cases compared with other methods, this demonstrates that *BCC*_*MOD*_ can break down the network quickly. Moreover, the largest S of *BCC*_*MOD*_ is the highest among all methods for all networks except Email and Router, which means *BCC*_*MOD*_ has the greatest damage to networks. From these results, in the point of network connectivity, *BCC*_*MOD*_ can quickly decompose networks and has the greatest damage to networks in most cases.

**Table 2:** The value of *p* corresponding to the largest *S*.

networks	B	Bc	J	R	*BCC*_*MOD*_
PowerGrid	0.2977	0.0597	0.2560	0.4974	0.0685
Lesmis	0.3216	0.5215	0.3960	0.8353	0.0784
Router	0.3737	0.1469	0.1002	0.0115	0.0137
Jazz	0.6070	0.5242	0.7036	0.9759	0.5148
Email	0.9325	0.8536	0.8169	0.9268	0.7467
Innovation	0.0011	0.0011	0.0011	0.0011	0.0011
Train	0.2213	0.3320	0.2049	0.7172	0.1844
Highschool	0.4693	0.4765	0.6065	0.7112	0.3357
Oz	0.7989	0.8940	0.9038	0.9288	0.5185

#### The size of giant component *σ*

Besides susceptibility index *S*, another metric, the size of giant component *σ* is used to evaluate the performance of methods. For details, sort edges descending order according to their score firstly, and then count the size of giant component *σ* after removing the edges from network one by one from high to low ranking scores.

**Figure 2:**
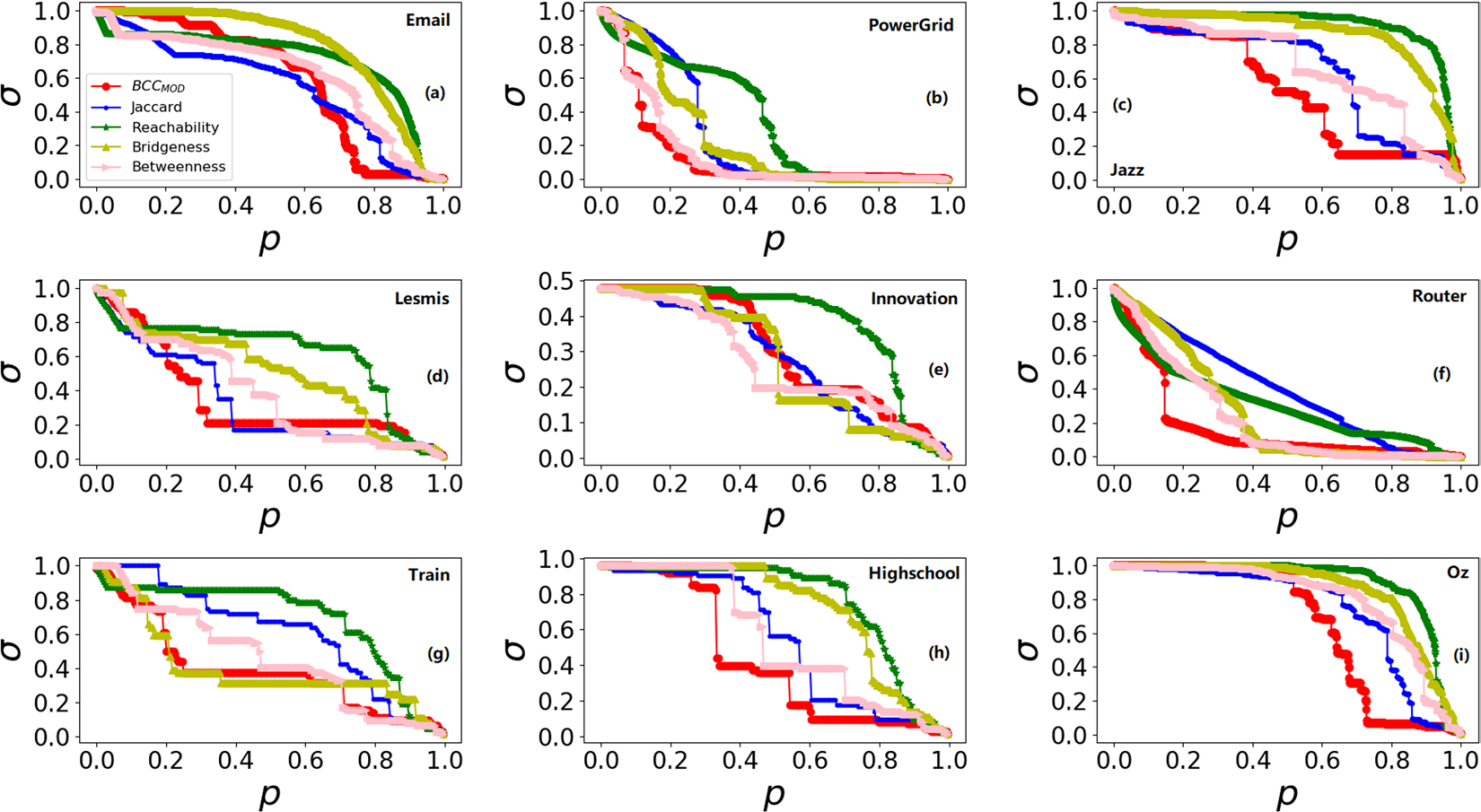
The size of giant component *σ* over different value of *p*.

The results are shown in Fig. 2. The faster the curve falls, the better the effect of method is. From Fig. 2(b,c,f,h,i), it can be found that the curve of *BCC*_*MOD*_ falls the fastest, which means *BCC*_*MOD*_ can break down the network quickly. And in Fig. 2(d,g), the falling speed of the *BCC*_*MOD*_ is close to the best case among all methods. In Fig. 2(a), the size of giant component *σ* drops quickly although it drops relative slow at the beginning. These results demonstrate that *BCC*_*MOD*_ can quickly decompose networks in most cases.

#### SIR model

In SIR model, there are three statuses: (1) *S*(*t*) denotes the number of nodes which may be infected (not yet infected); (2) *I*(*t*) denotes the number of nodes which have been infected and will spread the disease or information to susceptible nodes; (3) *R*(*t*) denotes the number of nodes which have been recovered from the disease or boredom the information and will never be infected by infected nodes again. In a network, each infected node will infect all susceptible neighbors with a certain probability *μ*. Infected nodes recover with probability *β* (for simplicity, *β* = 1 in this report) at each step. The process stops when there is no infected node. We can set a node to be infected and the others to be susceptible to estimate the influence of a single node in the network. The normalized final effected scale is defined as

3 $$F({t}_{c},u)=\frac{{n}_{R({t}_{c},u)}}{n},$$

where *n*_*R*_(*t*_*c*_, *u*) is the number of final effected nodes if node *u* is infected initially under SIR model and *F*(*t*_*c*_, *u*) is the finally normalized scale. To estimate the influence of edges, we can calculate the average influence of all nodes when remove a certain fraction of edges. We have an index

4 $${R}_{s}=\frac{{F}^{\mathrm{(1)}}({t}_{c})-{F}^{\mathrm{(2)}}({t}_{c})}{{F}^{\mathrm{(1)}}({t}_{c})},$$

where *F*^(*i*)^(*t*_*c*_) is the average final infected scale of all nodes, i.e., ${F}^{(i)}({t}_{c})=\frac{1}{n}{\sum }_{u\in V}F({t}_{c},u)$, and *F*^(1)^(*t*_*c*_) and *F*^(2)^(*t*_*c*_) are results of original network and the network after removing *p* of edges.

In Table 3, we show the spearman correlation coefficients between the ranking scores and the relative differences of real infected scale *R*_*s*_ with *μ*/*μ*_c_ = 2 where ${\mu }_{c}=\frac{\langle k\rangle }{\langle {k}^{2}\rangle -\langle k\rangle }$ in this report and all results are averaged over 200 independent implementations. Edges are descending order and divided into 50 parts. For each step only 1 part of edges (remaining other 49 parts) are removed and calculated the relative differences of real infected scale corresponding. Finally, two sequences (scores of the 2% edges and the relative differences of real infected scale) are obtained and the spearman correlation coefficients between them are obtained. From Table 3, it can be seen that *BCC*_*MOD*_ has maximal spearman correlation in PowerGrid, Lesmis, Router, Jazz, Innovation, Train and Email. These results demonstrate that the edge which *BCC*_*MOD*_ preferentially removed has a greater impact on the dissemination of real information.

**Table 3:** Spearman correlation coefficients between the ranking scores and the relative differences of real infected scale *R*_*s*_.

networks	B	Bc	J	R	*BCC*_*MOD*_
PowerGrid	0.3273	0.6425	0.1804	−0.2103	0.8406
Lesmis	0.3559	0.4416	0.1468	−0.1408	0.7024
router	0.5929	0.5914	−0.1241	−0.0561	0.8537
Jazz	0.1346	0.5526	0.4906	0.2034	0.7309
Email	0.3355	0.7077	0.5167	−0.1676	0.9232
Innovation	0.4767	0.7636	0.1284	0.1234	0.7523
Train	0.4832	0.5568	0.2256	−0.1013	0.7670
Highschool	0.7812	0.6267	0.4653	0.0613	0.7142
Oz	0.5650	0.8680	0.4653	0.1245	0.8324

All results are averaged over 200 independent implementations under *μ*/*μ*_*c*_ = 2.

**Figure 3:**
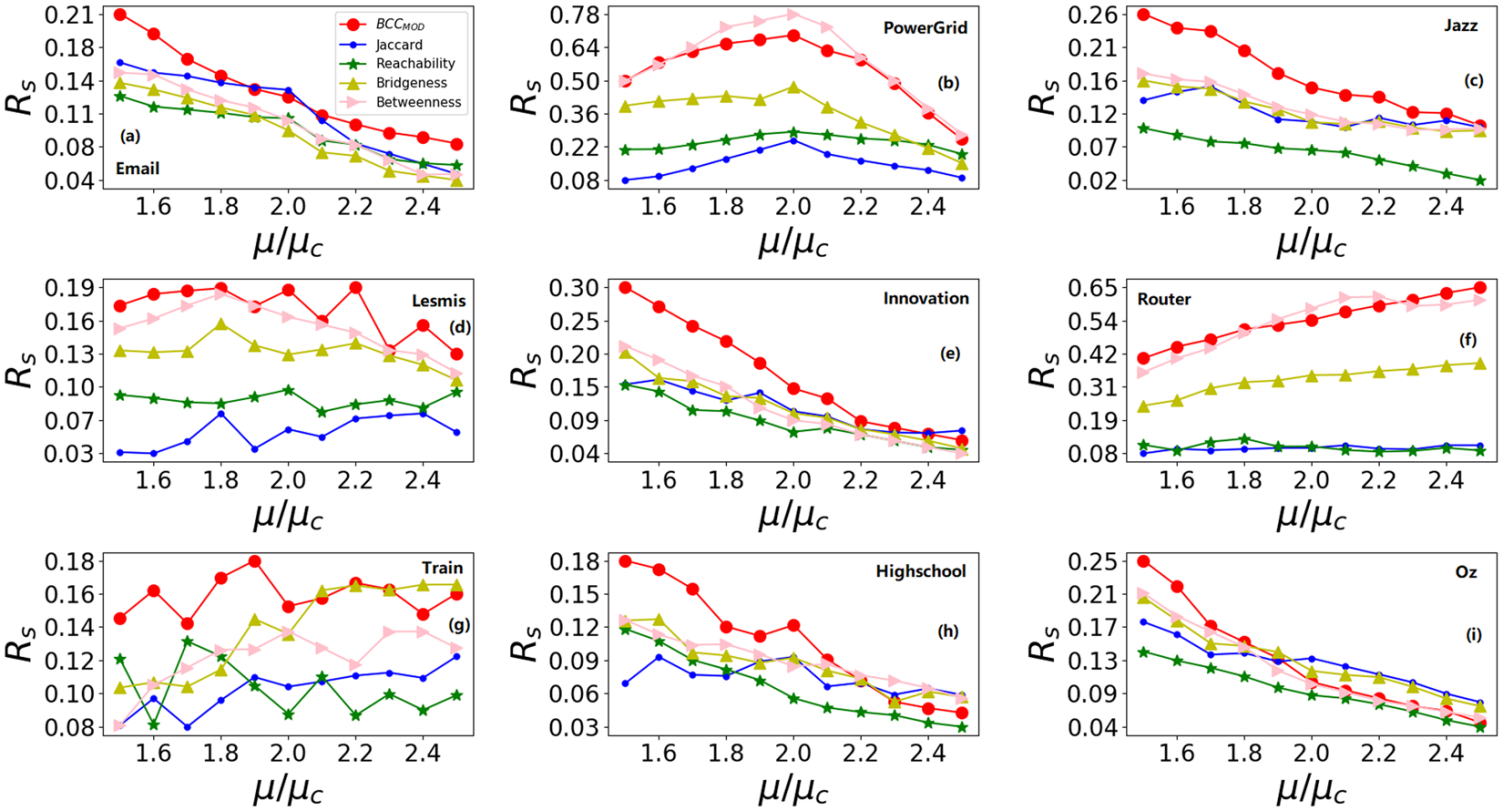
The relative differences of real infected scale *R*_*s*_ after removing top 5% ranking edges under different infect rates. All results are averaged over 100 independent implementations.

Figure 3 shows the relative differences of real infected scale *R*_*s*_ after removing top 5% ranking edges under different infect rates. It can be seen that *BCC*_*MOD*_ has higher *R*_*s*_ under different infect rates comparing with Jaccard, Bridgeness, Betweenness and Reachability methods. Generally, there is a significant impact on information spreading after removing top 5% ranking edges under *BCC*_*MOD*_.

**Figure 4:**
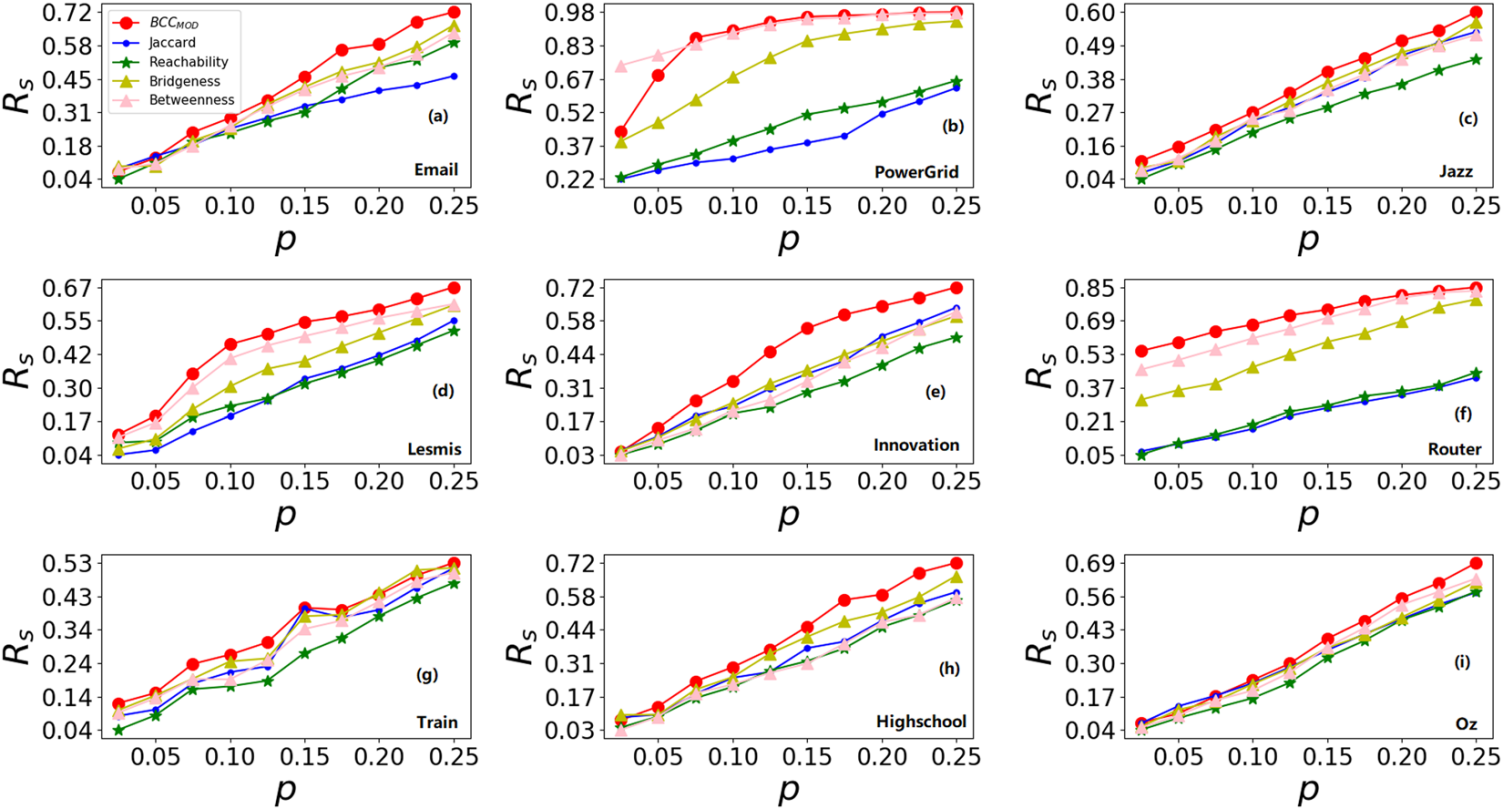
The relative differences of real infected scale *R*_*s*_ over each node as seed under different ratio of edges removing p. All results are averaged over 100 independent implementations under *μ*/*μ*_*c*_ = 2.

Figure 4 shows the relative differences of real infected scale *R*_*s*_ under different ratio of edges removing *p* with *μ*/*μ*_c_ = 2. From Fig. 4, it can be seen that *BCC*_*MOD*_ has higher *R*_*s*_ under different ratio of edges removing comparing with other methods. These results demonstrate that *BCC*_*MOD*_ has a greater impact on information spreading while removing a small part of edges than other methods.

## Discussion

In this report, the results show that if there are many different cliques containing both two related nodes of an edge, then the edge is not important for the perspective of spreading. We propose a global structural index, called *BCC*_*MOD*_ and compared with four well-known topological indices by susceptibility index *S*, the size of giant component *σ* and SIR model. The results show that *BCC*_*MOD*_ performs good in identifying critical edges both in network connectivity and spreading dynamic. As indicated by the experiments on the SIR model, *BCC*_*MOD*_ is effective in quantifying the spreading influences of edges. This will help us in some real-life applications such as controlling the spreading of diseases or rumors and withstanding targeted attacks on network infrastructures. What’s more, formal definitions of cliques have generally assumed that the network links are undirected, in directed networks, the definition of cliques will be modified^29,30^, correspondingly, the algorithm of mining critical edges also have subtle changes. Although the methods have a good performance, high computational complexity make it can’t be used in large-scale networks. In *BCC*_*MOD*_, all nodes’ degrees should be determined (running time is *O* (*m*)) and the time complexity for calculating the betweenness centrality of all edges in undirected networks is *O* (*mn*)^31^. The time complexity for finding all cliques in undirected networks is *O* (*M* (*n*)) where *M* (*n*) is the cost of multiplying two *n* × *n* matrices^32^ (for sparse matrices, *M* (*n*) is *O* (*n*^2^)). So the computational complexity of *BCC*_*MOD*_ is *O* (*mn* + *M* (*n*)) in undirected networks. *BCC*_*MOD*_ is a global index with not too high computational load and expected to be applied in small and middle undirected networks. How to optimization of our algorithm in large-scale networks and directed networks will be part of our future work. Besides SIR model, there also have other well-known dynamical processes to measure the importance of edges, for example, the susceptible-infected-susceptible (SIS) spreading model^33^ can examine how much information through the edge over a period of time.

## Methods

### Betweenness centrality

We know that betweenness centrality of edges indicates that the more the shortest paths between node pairs pass through the edge *e*(*u*, *v*), the more important the edge *e*(*u*, *v*) is. The betweenness centrality of an edge *e*(*u*, *v*)^15^ is defined as:

5 $$BC(u,v)=\sum _{s\ne t\in V}\frac{{\delta }_{st}(u,v)}{{\delta }_{st}},$$

where *δ*_*st*_ is the number of all the shortest paths between node *s* and node *t*, *δ*_*st*_(*u*, *v*) is the number of all the shortest paths between node *s* and node *t* which pass through the edge *e*(*u*, *v*), the larger the score *BC* is, the more important the edge is.

### Critical edge identification method

**Figure 5:**
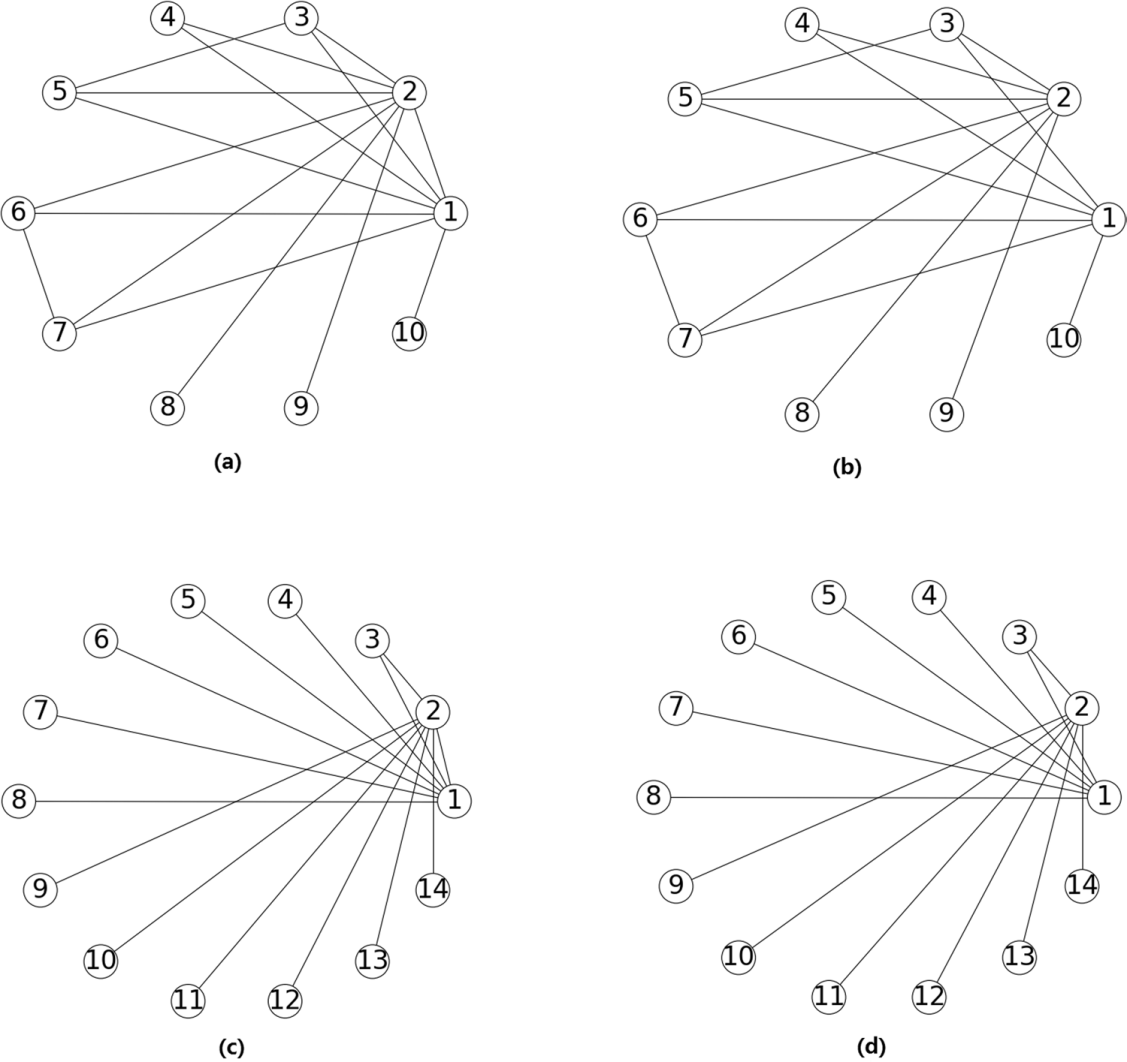
Four toy networks.

Generally, from the perspective of information spreading, the more important the two related nodes are, the more important the edge is. On the other hand, if there are many different cliques containing *e*(*u*, *v*), even *e*(*u*, *v*) is removed, the information also can spread from *u* to *v* (or *v* to *u*) easily through other edges in these cliques. Based on above 2 points and combined betweenness centrality of edges, a new index *BCC*_*MOD*_ (Betweenness Centrality and Clique Model)

6 $$BC{C}_{MOD}(u,v)=\frac{{k}_{u}{k}_{v}\cdot BC(u,v)}{\sum _{i=3}^{n}C{(u,v)}_{i}},$$

can be defined to measure the importance of an edge *e*(*u*, *v*). Where *BC*(*u*, *v*) is the betweenness centrality of edge *e*(*u*, *v*), *k*_*u*_ and *k*_*v*_ are the degrees of node *u* and node *v* respectively, *C*(*u*, *v*)_*i*_ is the number of cliques containing edge *e*(*u*, *v*) (in this report, clique means full connected subgraph, not the maximum full connected subgraph) whose size being *i*. For example *C*(*u*, *v*)_4_ = 3 means there are three cliques containing edge *e*(*u*, *v*) whose size being 4. In this method, the larger the score is, the more important the edge is. For example, as shown in Fig. 5(a,c), the degrees of nodes 1 and 2 are 7 and 8 respectively. In Fig. 5(a) (max size of cliques is 4), *C*(1, 2)_3_ is 5 and *C*(1, 2)_4_ is 2. When we remove edge *e*(1, 2), there are also many paths from node 1 to node 2, the effect of spreading is little. However, in Fig. 5(c) (max size of cliques is 3) with *C*(1, 2)_3_ being 1, when we remove edge *e*(1, 2), the effect of spreading is large since there is only one path (1, 3, 2) from node 1 to node 2. Table 4 shows the effect probability *p*_*e*_ of nodes 2, 3, and 9 with the original infected source being node 1 on SIR spreading model with full contact process. Taking node 2 as an example, in Fig. 5(a,b), its effect probability is 0.3733 and 0.2240 respectively under *μ* = 0.2. However, in Fig. 5(c,d), the effect probability of node 2 is 0.2392 and 0.0380 respectively under *μ* = 0.2.

**Table 4:** The ratio of infected cases among 10000 simulations of nodes 2, 3, and 9 with the original infected source being node 1 before and after edge *e*(1, 2) being removed in the toy network shown in Fig. 5 under different infected probability *μ*.

Fig. 4(a)				Fig. 4(b)			
Node	*μ* = 0.1	*μ* = 0.2	*μ* = 0.3	Node	*μ* = 0.1	*μ* = 0.2	*μ* = 0.3
2	0.1462	0.3733	0.6422	2	0.0490	0.2240	0.5036
3	0.1261	0.3001	0.5263	3	0.1152	0.2630	0.4408
9	0.0113	0.0743	0.1757	9	0.0053	0.0415	0.1269
**Fig.** 4(c)				**Fig.** 4(d)			
2	0.1121	0.2392	0.3637	2	0.0110	0.0380	0.0883
3	0.1094	0.2338	0.3833	3	0.0992	0.2010	0.3087
9	0.0108	0.0439	0.1095	9	0.0009	0.0072	0.0281

The Jaccard coefficient of an edge *e*(*u*, *v*) is defined as

7 $${J}_{e(u,v)}=\frac{|{{\rm{\Gamma }}}_{u}\cap {{\rm{\Gamma }}}_{v}|}{|{{\rm{\Gamma }}}_{u}\cup {{\rm{\Gamma }}}_{v}|},$$

where *u* and *v* are two related nodes of the edge *e*(*u*, *v*) and Γ_*u*_ is the set of *u*’s neighbors.The Bridgeness index of an edge *e*(*u*, *v*) is defined as

8 $${B}_{e(u,v)}=\frac{\sqrt{{S}_{u}{S}_{v}}}{{S}_{e(u,v)}},$$

where *S*_*u*_, *S*_*v*_ and *S*_*e*(*u*, *v*)_ is the size of max clique which contains node *u*, *v* and edge *e*(*u*, *v*), respectively.

The Reachability index of edge *e*(*u*, *v*) is defined as

9 $${R}_{e(u,v)}=\frac{1}{|V|}\sum _{s\in V}|R(s;{G}_{e(u,v)})|,$$

where |*V*| is the number of nodes, *G*_*e*_ is the subnetwork by removing an edge *e*(*u*, *v*) from original network and $|R(s;{G}_{e(u,v)})|$ is the number of reachable nodes from a node *s* over *G*_*e*_.
